# Targeting Accessories to the Crime: Nanoparticle Nucleic Acid Delivery to the Tumor Microenvironment

**DOI:** 10.3389/fphar.2018.00307

**Published:** 2018-04-04

**Authors:** Emily B. Harrison, Salma H. Azam, Chad V. Pecot

**Affiliations:** ^1^Center for Nanotechnology in Drug Delivery, UNC Eshelman School of Pharmacy, University of North Carolina at Chapel Hill, Chapel Hill, NC, United States; ^2^Curriculum in Genetics and Molecular Biology, University of North Carolina at Chapel Hill, Chapel Hill, NC, United States; ^3^UNC Lineberger Comprehensive Cancer Center, University of North Carolina at Chapel Hill, Chapel Hill, NC, United States; ^4^Division of Hematology/Oncology, University of North Carolina at Chapel Hill, Chapel Hill, NC, United States; ^5^Department of Medicine, University of North Carolina at Chapel Hill, Chapel Hill, NC, United States

**Keywords:** gene delivery, cancer, nanomaterials, immuno-oncology, cell targeting

## Abstract

Nucleic acid delivery for cancer holds extraordinary promise. Increasing expression of tumor suppressor genes or inhibition of oncogenes in cancer cells has important therapeutic potential. However, several barriers impair progress in cancer gene delivery. These include effective delivery to cancer cells and relevant intracellular compartments. Although viral gene delivery can be effective, it has the disadvantages of being immuno-stimulatory, potentially mutagenic and lacking temporal control. Various nanoparticle (NP) platforms have been developed to overcome nucleic acid delivery hurdles, but several challenges still exist. One such challenge has been the accumulation of NPs in non-cancer cells within the tumor microenvironment (TME) as well as the circulation. While uptake by these cancer-associated cells is considered to be an off-target effect in some contexts, several strategies have now emerged to utilize NP-mediated gene delivery to intentionally alter the TME. For example, the similarity of NPs in shape and size to pathogens promotes uptake by antigen presenting cells, which can be used to increase immune stimulation and promote tumor killing by T-lymphocytes. In the era of immunotherapy, boosting the ability of the immune system to eliminate cancer cells has proven to be an exciting new area in cancer nanotechnology. Given the importance of cancer-associated cells in tumor growth and metastasis, targeting these cells in the TME opens up new therapeutic applications for NPs. This review will cover evidence for non-cancer cell accumulation of NPs in animal models and patients, summarize characteristics that promote NP delivery to different cell types, and describe several therapeutic strategies for gene modification within the TME.

## Introduction

Over the last several decades, impressive advancements in nucleic acid delivery have brought these technologies to the clinic. Three gene therapies have been approved by the U.S. Food and Drug Administration (FDA), all of which rely on viral delivery systems. Non-viral systems are less immunogenic than viral systems, which may be required in cases where repeat administration is necessary, and they are non-mutagenic. While stable viral integration of genes may be a beneficial treatment for genetic disorders, a more transient regulation of gene expression may be preferred in other contexts. Non-viral nucleic acid delivery has not yet reached FDA approval, but several nanoparticles (NP)-based therapeutics are currently in clinical trials. For a thorough review of non-viral nucleic acid therapies in clinical development, we suggest (Yin et al., 2014). As these delivery platforms reach regulatory approval in the United States and elsewhere, they will pave the way for nucleic acid therapeutics in cancer and other diseases.

In parallel with advancements in nucleic acid delivery, the development of immunotherapies has revolutionized cancer treatment. Although historically cancer therapies have focused on directly killing cancer cells through chemotherapy and radiation, the success of immune checkpoint inhibitors and chimeric antigen receptor (CAR) T-cells has demonstrated that turning the immune tumor microenvironment (TME) against cancers can have strong therapeutic effects (Hoos, 2016). However, these immune-oncology drugs are only effective for subsets of patients, suggesting that additional factors are at play. An immune suppressive TME is one critical factor that can hamper T-cell invasion and anti-tumor effects. Taken together, harnessing NP-based nucleic acid delivery to the TME could transform a pro-tumoral and immuno-suppressive TME into a toxic environment for cancer cells. Here we review pre-clinical studies that demonstrate the feasibility of nucleic acid delivery to the TME for cancer therapy.

## Nanoparticles for Nucleic Acid Delivery

Naked nucleic acids display unfavorable biodistribution and pharmacokinetics: once injected into the blood stream, RNA and DNA can be quickly degraded by nucleases, phagocytosed by immune cells in the blood, or excreted through the kidneys. Therefore, to be effective, nucleic acids require delivery vehicles (Yin et al., 2014). All nucleic acids share a similar chemical structure: repeated nucleotides each composed of a five-carbon sugar linked to a nitrogenous base and connected by a phosphate backbone. While nucleic acids vary in size and contain either ribose or deoxyribose (in RNA and DNA, respectively), they are all negatively charged and hydrophilic. These properties allow them to be efficiently encapsulated into NPs. NPs are a diverse group of biomaterials that form structures in the nanometer scale. These include particles made of gold, silica, polymers, lipids, and others. While there are exceptions, lipids and polymers are the most common materials used for delivery of nucleic acids. For example, most commercially available transfection reagents use cationic lipids for effective intracellular delivery of DNA and RNAs such as mRNA, microRNA (miRNA) and short interfering RNAs (siRNAs) *in vitro*. In large part, *in vivo* nucleic acid delivery relies on similar principles but faces additional barriers such as stability in the circulation and delivery to target cells.

### Lipids

Lipid systems for *in vitro* gene delivery were first developed in the 1980s and were primarily composed of amphiphilic cationic lipids (Felgner et al., 1987). These molecules contain a polar head group, linker, and fatty acid chains that self-assemble into micellar, lamellar, or hexagonal structures in water: examples include N-[1-(2,3-dioleyloxy)propyl]-N,N,N-trimethylammonium chloride (DOTMA) and 1,2-Dioleoyl-3-trimethylammonium-propane (DOTAP). Incorporation of cholesterol and neutral lipids such as 1,2-Dioleoyl-sn-glycero-3-phosphoethanolamine (DOPE) can also increase stability and transfection efficiency. Permanently charged lipids result in toxicity, therefore ionizable systems have been developed. Ionizable lipids are positively charged in mildly acidic conditions where they can complex with nucleic acids; however, they remain uncharged at neutral pH which avoids systemic toxicity (Rietwyk and Peer, 2017).

### Polymers

Polymers can also be used to encapsulate nucleic acids for *in vivo* delivery. Generally, polymers can be divided into two groups: natural or synthetic. Biologically occurring molecules such as peptides, oligosaccharides, and even nucleic acids themselves are natural polymers. Synthetic polymers are chemically produced, such as poly(lactic-co-glycolic acid) (PLGA). Polymers can occur as a single repeating unit (homopolymers) or multiple unit types (copolymers). Additionally, polymers with discrete segments consisting of different repeating units, called block-copolymers can be made with a variety of useful properties. The cationic polymers poly-L-lysine (PLL) and polyethylenimine (PEI) were the earliest polymers used for condensing DNA. PEI has superior transfection efficiency and has been developed for *in vivo* and clinical delivery of nucleic acids (Yin et al., 2014). Combining PLGA, which is safe, biodegradable, and forms stable NPs, with PEI into mixed polymer NPs allows for effective gene delivery *in vivo* (Bivas-Benita et al., 2004). The neutral polymer polyethylene glycol (PEG) has also been used to reduce electrostatic interactions of both polymer and lipid-based NPs *in vivo* (Storm et al., 1995). Another nucleic acid delivery agent gaining popularity is chitosan, a natural co-polymer that can act as a biodegradable gene delivery agent (Wang et al., 2011). There are a wide variety of materials used in nanoformulations for nucleic acid delivery. The combination of these diverse materials and formulation techniques results in NPs with an array of sizes, charges, and surface properties. The properties of NPs used for nucleic acid delivery to the TME in preclinical studies are summarized in **Table 1**.

**Table 1:** Characteristics of nanoparticles used to target the tumor microenvironment in pre-clinical models.

Target cell type	Type of nucleic acid	Target gene(s)	Formulation material	Targeting moiety	Size (nm)	Surface charge (mV)	Reference
Macrophage	siRNA	*PPIB*	Amphipathic triblock copolymers (polymeric micelle)	Mannose	30	+20	Shann et al., 2013
	Cy5-labeled dsDNA	Na	Amphipathic triblock copolymers (polymeric micelle)	Mannose	nr	+20	Ortega et al., 2015
	siRNA	*IκBα*	Amphipathic triblock copolymers (polymeric micelle)	Mannose	nr	nr	Ortega et al., 2016
	siRNA and CpG oligonucleotide	*IL-10, IL-10RA*	PEGylated polymeric NP (glucan)	Galactose	270	nr	Huang et al., 2012
	miRNA	miR-155	PEGylated polymeric NP (polypeptide)	Galactose	100	+5	Liu L. et al., 2017
	siRNA	*CSF-1R*	PEGylated cationic phospholipid monolayer	ApoA 1-mimetic α-helical peptide linked to M2 macrophage binding protein	20	nr	Qian et al., 2017
	siRNA	*MIF*	Polymeric NP (glucan)	Glucan	nr	nr	Zhang et al., 2015b
	siRNA	*MIF*	Polymeric NP (glucan)	Glucan	80–120	nr	Zhang et al., 2015a
Monocyte	siRNA	*CCR2*	PEGylated cationic liposome	na	70–80	nr	Leuschner et al., 2011
Dendritic cell	siRNA	*PD-L1*	Polymeric NP (PEI)	na	nr	nr	Cubillos-Ruiz et al., 2009
	siRNA	*SOCS1*	Polymeric NP (PLGA-PLL)	na	150	–29	Heo et al., 2014
	siRNA	*STAT3*	Polymeric NP (PLGA-PLL)	na	100–200	–24	Heo and Lim, 2014
	siRNA or CpG oligonucleotide	*IL-10*	Polymeric NP (PLGA-PLL)	na	100–200	–20	Heo et al., 2015
	siRNA	*XBP1 or IRE1*	Polymeric NP (PEI)	na	nr	nr	Cubillos-Ruiz et al., 2015
	miRNA	miR-155	Polymeric NP (PEI)	na	nr	nr	Cubillos-Ruiz et al., 2012
Cancer-associated fibroblast	siRNA	*Wnt16*	PEGylated cationic liposome	Amino-ethyl anisamide	50	+25	Miao et al., 2015
	pDNA	soluble TRAIL	PEGylated cationic liposome	Amino-ethyl anisamide	70	+25	Miao et al., 2017b
	pDNA	PD-L1 and CXCL12 traps	PEGylated cationic liposome	Amino-ethyl anisamide	70	nr	Miao et al., 2017a
T cells	mRNA	megaTAL nuclease, *TREX2, Foxo1_3A_*	Polymeric NP (PBAE)	Anti-CD3 and anti-CD8 antibodies	110	+1	Moffett et al., 2017
Blood vessel endothelial cells	siRNA	*EZH2*	Polymeric NP (Chitosan-TPP)	na	100–200	+35	Lu et al., 2010
	Anti-miR	miR-132	Cationic liposome	αVβ3 ligand	100–200	nr	Anand et al., 2010
	miRNA	miR-200a and b	Polymeric NP (Chitosan-TPP)	RGD	100–200	nr	Pecot et al., 2013
	siRNA	*POSTN, FAK, PLXDC1*	Polymeric NP (Chitosan-TPP)	RGD	100–200	+40	Hee-Dong et al., 2010
	pDNA	*ATPμ-Raf*	Cationic liposome	αVβ3 ligand	100–200	+35	Hood et al., 2002
	siRNA	*CD31, Tie2*	PEGylated cationic liposome	na	100–200	nr	Santel et al., 2006
	siRNA	*VEGFR-1 and Dll4*	PEGylated lipid–polymer hybrid NP	na	100–200	0	Dahlman et al., 2014

*PEG, polyethylene glycol; PLL, poly-L-lysine; PEI, Polyethylenimine; TPP, tripolyphosphate; PLGA, poly(lactic-co-glycolic acid); PBAE, poly(*β*-aminoester); na, not applicable; nr, not reported.*

### NP Delivery to Tumors

**FIGURE 1:**
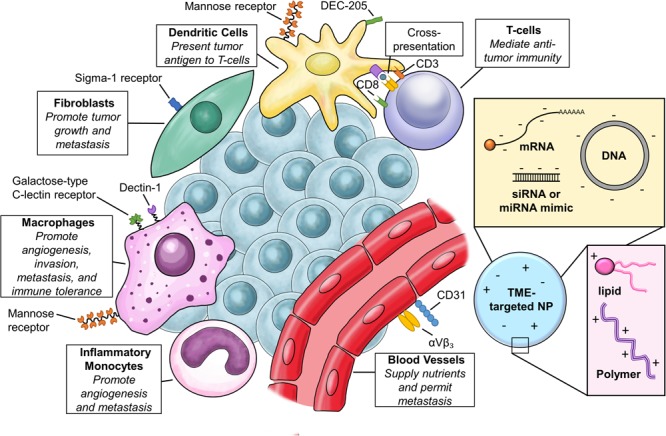
Targeting the tumor microenvironment (TME). Relevant cell types in the TME, common cell surface receptors for targeted delivery, and basic composition of nanoparticles (NPs) for TME targeting.

In the case of solid tumors, delivery to cancer cells is a formidable hurdle (Pecot et al., 2011), but evidence of tolerability and intracellular delivery has been demonstrated in phase I clinical trials for both lipid and polymeric NPs (Zuckerman and Davis, 2015). While leaky and inefficient vasculature can allow accumulation of NPs in the tumor (Prabhakar et al., 2013), solid tumors also have stroma that contains a vast milieu of non-cancerous constituents that include fibroblasts, tumor-associated macrophages (TAMs), endothelial cells and extracellular matrix components (ECM) that additionally impair access to the tumor parenchyma. In the case of NPs loaded with small molecule drugs, delivery to TAMs in the TME may be beneficial for local and sustained release of drug (Miller et al., 2015). This is not the case for nucleic acid delivery, where not only is delivery to appropriate cells required, but the payload must also reach key intracellular compartments. While strategies to allow better penetrance of NPs through the tumor stroma are being explored, caution is warranted. Disruption of tumor stroma may remove important elements of nutrition and growth factors, but it can also promote resistance (Miao et al., 2015). One alternative strategy is to target tumor-associated cells within the TME for cancer therapy. Commonly found in the tumor periphery, these cells are the first to encounter NPs as they leave the circulation. Many cell types within the TME also express unique cell surface markers, which can be utilized for targeted delivery. Given the influence of TME cells on all of the hallmarks of cancer, this is an enticing direction to pursue (Hanahan and Coussens, 2012). TME cell types, their role in cancer biology, and surface markers commonly used to target them are summarized in **Figure 1**.

## Types of Nucleic Acids for NP Delivery

### DNA

Delivery of exogenous DNA offers a great therapeutic opportunity for cancer. One approach is overexpression of genes that can suppress or kill tumor cells. These genes can be human in origin, viral proteins, bacterial toxins, or proteins designed for desired functions. One such example is NP delivery of the gene encoding anti-tumoral viral protein E1A, which was successfully delivered and expressed in humans (Chang et al., 1997; Yoo et al., 2001; Ueno et al., 2002). Many barriers to gene delivery are universal to all oligonucleotide species: stability in circulation, cell uptake, and endosomal escape. DNA must additionally be delivered to the nuclear compartment to permit access to transcriptional machinery. It was shown over 30 years ago that DNA microinjected into the nucleus, but not the cytosol, produces a gene product (Capecchi, 1980). Improved delivery systems and alterations in DNA sequences can enhance nuclear delivery. For example, import into the nucleus can be aided by inclusion of a nuclear localization signal (NLS) in the plasmid DNA (pDNA) sequence (Brandén et al., 1999; Zanta et al., 1999). Also, strong viral or eukaryotic promoter sequences can be added to activate transcription (Capecchi, 1980). Plasmids can be further tailored by using tissue-specific promoters to reduce potential off-target effects (Gorski et al., 1986).

Important safety concerns for DNA delivery are immune stimulation and risk of insertional mutagenesis. The risk of insertional mutagenesis is much higher for viral delivery systems, but cannot be excluded for episomal pDNA (Glover et al., 2005; Baum et al., 2006). Like other oligonucleotides, DNA can stimulate immune responses that should be considered when designing therapeutics. Immune responses to DNA are caused by unmethylated CpG motifs which stimulate B-cell proliferation and cytokine release through TLR9 (Krieg et al., 1995; Klinman et al., 1996; Hemmi et al., 2000). The robust immunostimmulatory effect of CpG DNA makes it a potentially useful vaccine adjuvant (Gurunathan et al., 2000). Alternatively, the immune effects of CpG can also be removed by altering the DNA sequence to replace CpG with CpG-S motifs (Krieg et al., 1998). Thorough reviews of DNA delivery technology platforms have been published elsewhere (Nishikawa and Huang, 2001; Yin et al., 2014).

Delivery of DNA encoding a gene of interest is a powerful tool for gene therapy and important advances toward this goal have been made. While no therapies have been approved by the FDA thus far, several non-viral DNA strategies are being evaluated clinically, including in clinical trials for cancer therapy. In a phase 1 clinical trial, the tumor suppressor gene *TUSC2* was delivered to lung cancer patients using DOTAP-cholesterol liposomes, resulting in transgene expression and activation of apoptotic pathways (Lu et al., 2012). This therapy is now in phase 1/2 trials. Other gene delivery trails include IL-12 gene delivery in PEG–PEI–cholesterol NPs to enhance immune response (Kendrick et al., 2008; Anwer et al., 2010) and co-delivery of two tumor suppressor genes somatostatin receptor subtype 2 (SSTR2) and deoxycytidine kinase::uridylmonophosphate kinase (DCK::UMK) complexed to PEI (Buscail et al., 2015). Delivery of the cytotoxic diphtheria toxin A gene as a “suicide” gene under cancer specific promoters in PEI NPs is also being evaluated in ovarian, pancreatic, and bladder cancer and has demonstrated good safety profiles and anti-tumor efficacy (Sidi et al., 2008; Smaldone and Davies, 2010; Hanna et al., 2012; Gofrit et al., 2014). If these non-viral DNA therapeutics show substantial efficacy in clinical trials, they will pave the way for non-viral DNA in cancer therapy and other diseases. After 3 decades of technology development, therapeutic DNA delivery in humans is becoming a reality.

### mRNA

The goal of mRNA delivery is the same as DNA delivery, to deliver a therapeutic gene that will be translated into protein within target cells. In contrast to DNA, mRNA needs to reach the cytosol and be recognized by ribosomes. Transfection efficiency with mRNA is higher than for DNA, especially in non-dividing cells (Yamamoto et al., 2009). Also, mRNA delivery does not pose the risk of insertional mutagenesis. Although stability of naked mRNA is poor, chemical modifications and protection from serum endonucleases in NP delivery vehicles can increase mRNA stability. As with DNA, exogenous mRNA can also stimulate an immune response through TLR3, TLR7, TLR8, and retinoic acid receptor responder protein 3 (RARRES3 or RIG-I) (Yin et al., 2014). Chemical modifications can reduce recognition of mRNA by the immune system (Karikó et al., 2005).

The structure of mRNA is critical for recognition by the eukaryotic translation machinery. At the core of the mRNA structure is an open reading frame (ORF) that is translated into protein. Flanking the ORF are two untranslated regions (UTRs) at the 3′ and 5′ ends that allow for regulation of translation. Finally, a 5′ methyl cap and a 3′ poly adenosine tail book-end the mRNA and are necessary for efficient translation (Gallie, 1991). Increasing the poly(A) tail length also improves stability (Holtkamp et al., 2006). Commercial kits are available to synthesize mRNA with all necessary structural components from plasmid DNA, though optimization for target cell types can also help improve translation efficiency (Yamamoto et al., 2009).

There are over a dozen clinical trials using mRNA for vaccines, adjuvants, or to express antigens either in dendritic cells *ex vivo* or by direct injection. However, gene replacement therapy is still in pre-clinical development (Kaczmarek et al., 2017).

### MicroRNAs (miRNAs)

Discovered in 1993, miRNAs are a class of non-coding, regulatory RNAs that have critical roles in nearly all biological processes, including cancer. miRNAs can serve as both oncogenes and tumor suppressors (Farazi et al., 2013). Primary miRNA transcripts have characteristic hairpin structures that are recognized and processed by RNase III enzyme Drosha, which produces a stem loop precursor miRNA (pre-miRNA) of ∼70 nucleotides (Lee et al., 2003). Final cleavage by Dicer results in a mature dsRNA (Hutvágner et al., 2001). The mature ∼22 nucleotide miRNA associates with the RNA-induced silencing complex (RISC), and one or both strands of the duplex guide RISC to complementary sequences within target mRNA. Strand selection by RISC is likely based on stability. The two strands are named 5p and 3p corresponding to the 5′ and 3′ ends of the miRNA precursor hairpin, respectively. Target sequences complimentary to the 2–7 nucleotide “seed” region of the miRNA sequence are frequently found in the 3′ UTR of mRNAs, but can also be within coding or intronic regions. Binding of miRNA to target mRNA results in degradation or destabilization of the mRNA and can also cause translational repression (Ha and Kim, 2014). Generally speaking, each miRNA can target hundreds of unique mRNAs, and thus can regulate transcriptome-wide changes. In this way, miRNAs are critical regulators of cell identity and state (Kosik, 2010). Additionally, miRNAs are essential for immune cell development and immune activation (Xiao and Rajewsky, 2009) as well as cross-talk between cancer cells and the TME (Chou et al., 2013).

To replace a downregulated miRNA, synthetic double-stranded RNAs (dsRNAs) carrying the same sequence as the endogenous mature or precursor miRNA can be used. These miRNA “mimics” are smaller and more stable than mRNA, allowing ease of encapsulation in several types of NPs. Chemical modifications to miRNAs can be made in the same way as mRNAs or siRNAs to increase stability and reduce inflammatory response. Nucleic acid based inhibitors of miRNAs include locked-nucleic acids (LNAs), antagomirs, anti-miRs, and miR-sponges have been characterized in more detail elsewhere (Ling et al., 2013). Therapeutic strategies modulating miRNA function are already in clinical trials, and a comprehensive review of miRNA therapeutics is provided elsewhere (Rupaimoole and Slack, 2017). Inhibiting miR-122, a critical player for hepatitis infection is being investigated in multiple clinical trials, and inhibition of miR103/107 is in clinical trials for alcoholic fatty liver disease. Both drugs utilize unencapsulated anti-miRs. While these therapeutics target the liver, a relatively accessible organ target for nucleic acid delivery, trials are also in progress for cancer therapy. Delivery of miR-16 with EGFR-targeted EnGeneIC Delivery Vehicle nanocells completed phase 1 clinical trials in mesothelioma with an acceptable safety profile and signs of efficacy (van Zandwijk et al., 2017). In contrast, phase 1 trials for miR-34 mimics for multiple solid tumors were recently terminated due to severe immune-related and marrow suppressive adverse events. A 110 nm liposomal carrier composed of ionizable lipids was used in these trials. The precise source of the inflammatory reactions—be it due to the carrier, miRNA mimic, or synergy between the two—is not known (Beg et al., 2017; Rupaimoole and Slack, 2017). It is clear that going forward, extensive pre-clinical evaluation of immune stimulation by miRNA-directed therapies must to be an important consideration.

### siRNA

siRNAs are ∼21 nt dsRNAs that interact in the cytoplasm with the RISC complex to degrade target mRNAs. In structure, siRNAs are identical to miRNAs and can be modified and encapsulated in the same way. Other than the fact that miRNA mimics are based on endogenous genes while siRNAs are synthetically designed, the primary difference between miRNAs and siRNAs is the sequence specificity. Instead of a miRNA seed region of 6–8 nt with complementarity to the 3-UTR of target mRNAs, by design siRNAs usually have 100% complementarity to target mRNAs. As such, siRNAs have potent activity on a single target. In contrast, miRNAs have the potential to target hundreds of mRNAs, but generally this inhibition is to a lesser extent. The targets of siRNAs are degraded by the endonuclease activity of Argonaut 2 associated with the RISC, while miRNAs usually cause deadenylation or translational repression of target mRNAs by association with other Argonaut complexes devoid of nuclease activity. Therefore, the pharmacodynamics of miRNA mimics and siRNAs have important differences. However, the pharmacokinetics and biodistribution of these two oligonucleotides is the same, since their chemical structure is identical. Another consideration is stimulation of immune responses by exogenous RNA. For instance, siRNAs within liposomal or polymeric NPs can induce an inflammatory cytokine response that is greater than either component alone. The response is mediated by TLRs and is sequence dependent, with GU-rich sequences inducing the most potent cytokine response (Judge et al., 2005). Modifications of RNA, such as incorporation of 2′-O-methyl nucleosides, can reduce inflammatory response without decreasing gene silencing (Judge et al., 2006). Inflammatory cytokine responses have been observed in siRNA and miRNA clinical trials, and therefore evaluating immunostimulation should be a concern when translating these therapeutics to the clinic. Currently, dozens of clinical trials of siRNA-based therapies have been completed or are ongoing, for in depth review see Wittrup and Lieberman (2015) and Zuckerman and Davis (2015).

### Other Non-coding RNAs

Several classes of non-coding RNAs have been identified including piwi-interacting RNAs, endogenous siRNAs, long-non-coding RNAs, and circular RNAs. The function of these species and their role in disease biology are being actively investigated (Esteller, 2011). As the role of non-coding RNAs in cancer biology continues to unfold, therapeutic approaches to modulate them will be of increasing interest (Gutschner and Diederichs, 2012). Therapeutic delivery of non-coding RNAs will face similar challenges to other types of RNA, such as stability and uptake into appropriate cells and intracellular compartments.

### Genome Editing

The targeted manipulation of genomic DNA in living cells is possible through the use of engineered nucleases, such as mega nucleases, zinc finger nucleases (ZFN), transcription activator-like effector-based nucleases (TALEN), and the clustered regularly interspaced short palindromic repeats (CRISPR)-Cas system (Cox et al., 2015). The enthusiasm surrounding genome editing technology is reminiscent of that generated by the discovery of RNAi (Fire et al., 1998). However, similar hurdles impede therapeutic translation. To enact genome editing *in vivo* nucleases must be present within target cells. While delivery of protein may be possible, most approaches utilize delivery of mRNA or DNA encoding nucleases. Using viral vectors, such as AAV, to deliver nucleases raises additional concerns. Integration of nuclease DNA leads to constitutive expression of nucleases capable of genome editing and increases the chances of off-target mutations. For this reason, transient expression – as is achieved with NP systems-may be preferable.

CRISPR-Cas9 is a highly specific gene editing tool that is rapidly becoming a standard lab technique. In its simplest embodiment, it requires the presence of a Cas9 protein and single guide RNA (sgRNA) to be present in the same cell (Liu C. et al., 2017). The first use of CRISPR-Cas9 in clinical trials has already taken place. In this trial the T-cell exhaustion receptor PD-1 was genetically removed *ex vivo* by CRISPR-Cas9 technology and cells were then delivered to the patient (Cyranoski, 2016). Similarly, CRISPR-Cas9 is being used to remove the CCR5 receptor from hematopoietic stem cells as a therapeutic strategy for HIV. Since HIV enters cells through CCR5, removing this receptor is expected to reduce the ability of HIV to infect transplanted cells (Li et al., 2015). Application of this technique to other cell-based therapies could soon follow. It will certainly be more challenging to edit cells *in vivo* but several groups are working toward this important goal, including developing strategies for cancer treatment (Sánchez-Rivera and Jacks, 2015).

## Targeting Cells in the TME

### Mononuclear Cells

#### Macrophages

Tumor-associated macrophages (TAMs) are often pro-tumoral by promoting angiogenesis, invasion, metastasis, and immune tolerance (Noy and Pollard, 2014). Given these and other important roles in tumor progression, there is a growing interest in targeting TAMs as a cancer therapeutic. Macrophages are often characterized by the balance of pro- and anti-inflammatory characteristics as “M1” or “M2” macrophages, respectively. While in most cases this terminology is an oversimplification, it is a useful reference to describe macrophage subsets. M1 macrophages are characterized by high levels of major histocompatibility complex (MHC) class II molecules, pro-inflammatory cytokines and inducible nitric oxide synthase 2 (Nos2). Conversely, M2 macrophages express low levels of these markers and instead express high levels of arginase-1 and scavenger receptors like the mannose receptor (MR) (Martinez et al., 2009). Initial *in vitro* studies suggested macrophages could kill tumor cells, however, in the TME secreted factors shift TAMs to a pro-tumoral M2 phenotype (Sica et al., 2006). Therefore, reprograming TAMs to an M1-like state could reverse the pro-tumoral effects. We review here characteristics of NPs that promote macrophage uptake and detail studies utilizing NP gene delivery to target TAMs for cancer therapy.

Most NP platforms are in the size range of viruses (20–250 nm) and bacteria (0.2–10 μm), and consequently they are readily taken up by phagocytic cells of the immune system, such as macrophages and dendritic cells. In fact, uptake by the mononuclear phagocytic system (MPS), also called the reticuloendothelial system (RES), has been considered a critical obstacle to NP drug delivery. One example of this is the first FDA approved NP drug Doxil, a nanoliposome formulation of doxorubicin. Doxil was coated with a hydrophilic neutral polymer, PEG, to reduce its recognition by the MPS, creating a “stealth” effect (Working et al., 1994). Despite these modifications, clearance by macrophages still has a major influence on Doxil pharmacokinetics (La-Beck et al., 2012). The proclivity of macrophages for taking up NPs can even be utilized to image macrophages *in vivo* (Weissleder et al., 2014). Interestingly, M2 macrophages take up both 300 nm PEG hydrogel NPs and 30 nm quantum dots at higher rates than M1 macrophages due to increased levels of scavenger receptors such as MR, suggesting that TAMs may be especially sensitive to NP delivery. This phenomenon does not extend to microparticles, as 6 μm PEG hydrogels are not affected by macrophage polarization (Jones et al., 2013). Additionally, single cell pharmacokinetics of NPs within tumors has revealed that TAMs can serve as reservoirs for NPs, releasing small molecule drugs (Miller et al., 2015). The fate of oligonucleotide payloads in macrophages is less certain, although there is some evidence that macrophages may also transfer genes to cells at sites of inflammation (Haney et al., 2013; Mahajan et al., 2016). Because much effort has gone into reducing phagocyte clearance of NPs, there is a wealth of studies detailing the characteristics that reduce and consequently those that enhance uptake into this population. Detailed review of factors influencing macrophage NP uptake is covered elsewhere (Gustafson et al., 2015).

Macrophage NP uptake can occur through micropinocytosis, phagocytosis, and receptor-mediated endocytosis (Gustafson et al., 2015). Factors influencing uptake include charge, size, and surface chemistry. A positive surface charge facilitates uptake by many types of cells having a negative membrane potential. In contrast, greater net charge in either the positive or negative direction increases uptake of NPs by macrophages. The uptake of chitosan NPs with charges ranging from -40 to +35 was examined for murine macrophages. In these cells, increasing charge in both the negative and positive direction increased macrophage uptake, while non-phagocytic cells more efficiently took up positively charged particles. NP size is also a key determinant: large NPs 300–500 nm in size are taken up more efficiently than 150 nm particles by murine macrophages (He et al., 2010). In addition, uptake of particles by macrophages is highly dependent on serum protein adsorption. PEGylation can decrease, but not eliminate, protein adsorption and macrophage uptake (Xie et al., 2007; Walkey et al., 2012). These factors are important for non-targeted or passive uptake; however, further cell-type specificity can be achieved with targeting moieties.

Ligands or antibodies to cell surface receptors can be used to decorate the NP surface and enhance macrophage uptake. Receptors that mediate macrophage NP uptake include folate receptor (FR), MR, cluster of differentiation 163 (CD163), Legumanin, galactose-type C-type lectins, and cluster of differentiation 11b (CD11b) (Binnemars-Postma et al., 2017). Mannose is one of the most common macrophage targeting ligands, but MR (also known as CD206) is also present on other phagocytes, such as DCs (McKenzie et al., 2007), which could result in off-target effects. However, MR is upregulated in M2-like TAMs with decreased MHC II expression (Movahedi et al., 2010). Additionally, CCR2 knockout mice that have fewer TAMs have reduced tumor uptake of MR-targeted nanobodies, indicating that MR binding is through CCR2 derived cells, including macrophages (Movahedi et al., 2012). Mannosylated polymeric micelles are able to deliver siRNA and mediate TAM gene silencing *in vitro* and *in vivo* (Shann et al., 2013; Ortega et al., 2015). The galactose-type C lectin receptor has also been targeted for macrophage nucleic acid delivery by attaching its ligand, galactose, to the surface of NPs (Huang et al., 2012; Liu L. et al., 2017). One sophisticated approach utilized a dual targeting moiety: an apolipoprotein A1 mimetic (α-peptide) served as a ligand for SR-1B and was linked to a M2 macrophage binding protein (M2pep) to deliver NP-encapsulated siRNAs (Qian et al., 2017). Macrophages also express receptors capable of recognizing a variety of pathogen-associated molecular patterns (PAMPs) and incorporation of PAMPs into NP design can facilitate NP uptake by TAMs. For example, nanocomplexes incorporating glucan, a PAMP associated with fungi, were shown to target TAMs (Zhang et al., 2015a,b). In summary, TAMs act as natural sinks for NPs. Further targeting with receptor specific ligands or antibodies can facilitate uptake, but complete discrimination between macrophages and other mononuclear cells has not been clearly demonstrated.

A small number of studies have shown efficacy of nanoparticle nucleic acid delivery to target and reprogram TAMs for cancer therapy. In a melanoma mouse model, delivering anti-CSF-1R siRNA targeted to TAMs reduced tumor growth by 87% and prolonged survival. Non-targeted particles also inhibited tumor growth, but not as dramatically. This therapeutic effect corresponded with decreased immunosuppressive cytokines IL-10 and TGF-β, and increased immunostimmulatory cytokines IL-12 and IFN-γ as well as increased the function of CD8+ T-cells (Qian et al., 2017). Similarly, pro-inflammatory miR-155 was delivered in redox and pH sensitive NPs targeted with galactose moieties to the macrophage galactose-specific C-type lectin receptor. Galactose targeting increased *in vitro* miR-155 uptake in TAMs, but not B16-F10 cells. Delivery of miR-155 NPs increased IL-12 and MHCII positive cells, as well as decreased M2 markers. Increased numbers of activated T-cells and NK cells were observed, and anti-tumoral effects were elicited (Liu L. et al., 2017). Intratumoral injection of modified glucan nanocomplexes carrying siRNA has also been shown to effectively inhibit gene expression in macrophages. Delivery of siRNA to migration inhibitory factor (MIF) in glucan NPs reduced both released and intracellular MIF in TAMs and in cancer cells. This resulted in reduced M2 markers and increased inflammatory cytokines TNF-α and IL-2. This treatment also increased CD4+ and CD8+ cells in the tumor and promoted anti-tumor immunity (Zhang et al., 2015a,b). These reports support that oligonucleotide delivery can be used to reprogram TAMs from an M1 to M2 phenotype to promote anti-tumor effects.

#### Inflammatory Monocytes

Inflammatory monocytes (IMs) can give rise to TAMs and other myeloid suppressor cells which promote angiogenesis and subsequent metastasis. Recruitment of IMs relies on the chemokine CCL2 (Qian et al., 2011). Blocking this axis with receptors against CCL2 or its cognate receptor CCR2 has been the subject of clinical trials, but pharmacological inhibition of this axis has proved challenging in part due to rebound effects (Lim et al., 2016). An alternative strategy used screening approaches to identify both optimal lipids and siRNA sequences for inhibition of CCR2 in monocytes, no targeting ligands were used. In a lymphoma model, inhibition of CCR2 in monocytes reduced tumor size and number of TAMs. This therapy also inhibited expression of VEGF and reduced microvessel density in the tumors (Leuschner et al., 2011). Whether NP-based targeting of the CCL2-CCR2 axis can evade withdrawal effects seen with antibody targeting of the CCL2-CCR2 axis remains to be seen. However, the ability to silence genes in monocytes has demonstrated clear therapeutic potential.

#### Dendritic Cells

As part of the innate immune response to pathogens, dendritic cells recognize foreign materials through pattern recognition receptors (PRRs) or compliment binding leading to phagocytosis. Inside dendritic cell lysosomes, processing of pathogenic proteins results in the generation of peptide fragments that are presented on MHC receptors to be recognized by members of the adaptive immune system. Cross presentation of antigens from DCs to CD8^+^ T-cells is required for anti-tumor immunity. As such, DCs are the primary targets of cancer vaccines and their actions are required for effective cytotoxic T-cell response in checkpoint blockade inhibitor therapies. NPs from 20 nm to 3 μm are readily taken up by dendritic cells, presumably due to the size similarity to viral and bacterial pathogens. The size of NPs and their ability to present multivalent antigens clearly points to vaccine applications (Bachmann and Jennings, 2010). For these reasons an increasing number of NP-based vaccines with or without additional immune agonists are being designed for cancer therapy (Mizrahy et al., 2017). Here we will consider those that additionally incorporate nucleic acid delivery. A smaller number of studies have utilized gene delivery to modulate the activation of dendritic cells and subsequent cross-presentation to T-cells.

Surface coating can also increase DC NP uptake. For example, natural coatings such as mannosylation or glycosylation increase DC uptake through interactions with MR (Jiang et al., 2015; De Coen et al., 2016; Wang et al., 2016). However, as previously described, MRs are also expressed on macrophages which may compete with DCs for NP uptake (Stahl and Ezekowitz, 1998). Alternatively, antibody-based targeting has also been reported. One example is a clinical trial using targeting antibodies against MR to deliver a peptide antigen to APCs. This therapy induced humoral and T-cell responses in melanoma patients (Morse et al., 2011). Reports indicate that antibodies against DEC-205 can also enhance DC uptake and increase downstream immune activation relative to non-targeted NPs (Raghuwanshi et al., 2012; Walters et al., 2015). Using antibodies against DEC-205 fused with a tumor antigen induced humoral and cellular immunity in patients with advanced malignancies (Dhodapkar et al., 2014). While MR and DEC-205 are commonly used for NP or vaccine targeting, other targets have also been tested (Sehgal et al., 2014a), including targets that enhance uptake in subsets of DCs (Sehgal et al., 2014b). However, in some cases the material composition of the NP carrier may be more important than targeting ligands. For instance, lipid based “nanogels” were more readily internalized than PLGA NPs (Look et al., 2014). Also, linear PEI nanocomplexes were more effective than anti-CD11c antibody-targeted zwitterionic liposomes at siRNA delivery to DCs (Cubillos-Ruiz et al., 2009).

Several reports both *in vitro* and *in vivo* have shown that delivering siRNAs in addition to antigenic peptides and adjuvants to DCs can further enhance anti-tumor immune responses. Heo et al. (2014) used polymeric micelles to deliver a tumor antigen and siRNA for the immunosuppressive Suppressor of Cytokine Signaling 1 (SOCS1) to dendritic cells *in vitro*. Delivery of SOCS1 siRNA increased secretion of pro-inflammatory cytokines by cultured DCs and activation of T-cells by cross presentation (Heo et al., 2014). Two additional studies by Heo et al. (2014) examined *in vivo* efficacy of NP siRNA to DCs. In one study, the investigators formulated multifunctional polymeric NPs carrying tumor model antigen OVA, dendritic cell activator imiquimod (R837), and siRNAs for STAT3. The immune activation induced by R837 is inhibited by STAT3, so the authors hypothesized that this combination would produce a more robust activation of DCs. PLGA (R837/STAT3 siRNA) NPs were taken up efficiently by DCs, elicited cytokine response, antigen cross-presentation, and trafficking of DCs to draining lymph nodes when injected *in vivo*. Furthermore, incorporation of STAT3 siRNA significantly increased anti-tumor immunity (Heo and Lim, 2014). In a different approach, tumor bearing mice were first treated with hyaluronic acid (HA) and paclitaxel (PTX) complexes to induce immunogenic cell death. This treatment was followed by administration of NPs containing CpG adjuvant and IL-10 siRNAs. IL-10 is an immunosuppressive cytokine and its inhibition further enhanced the immune response. These multifunctional NPs trafficked to draining LNs and promoted antitumor immunity *in vivo* (Heo et al., 2015).

In ovarian cancer tumor-associated DCs (tDCs) have a particularly tolerogenic role (Huarte et al., 2008). By inhibiting tolerogenic pathways in DCs with siRNA, therapeutic benefits were observed in ovarian cancer models. In addition to siRNA, miRNA can also be delivered to tDCs to induce anti-tumor immunity. For example, delivery of siRNAs against members of the ER stress pathway, XBP1 and IRE1, which inhibit cross presentation in tDC through lipid accumulation. NP delivery of XBP1 or IRE1 siRNA reduced metastasis and increased survival in ovarian cancer models. Importantly, this phenomenon was ablated in Rag2 deficient mice suggesting that immune and not direct cancer targets were responsible (Cubillos-Ruiz et al., 2015). miR-155 is considered to be an oncogenic miRNA, however, it is also necessary for cross-presentation of DCs. Using PEI NPs, delivery of miR-155 mimics produced potent anti-tumor effects with about 33% of mice showing no disease progression 80 days after controls had succumbed to disease. This anti-tumor effect was accompanied by transcriptome wide changes in tDCs, highlighting the utility of miRNAs for reprograming the TME (Cubillos-Ruiz et al., 2012). Interestingly, the authors also found that a bulged dsRNA that required processing by RNase enzyme DICER was most effective at gene silencing. Another aspect of the studies by Cubillos-Ruiz et al. (2009, 2012, 2015) was an immune-stimulatory effect of PEI NPs containing even non-targeting RNA through TLR pathways. Overall, RNA delivery to tDCs has been shown to be an effective therapeutic strategy in mouse models of ovarian cancer.

Historically, vaccines have relied on peptide antigens, but an alternative vaccine strategy is delivery of DNA or mRNAs. In brief, DNA or mRNA encoding an antigen are injected, the genetic material is taken up by cells at the injection site, and then translated into protein. Proteins encoded in the DNA or mRNA can be expressed in myocytes or keratinocytes at the injection site and are subsequently recognized by APCs or directly taken up by DCs followed by internal processing and presentation. DNA vaccines are currently used in veterinary medicine, but have thus far not been successfully translated to humans (Rice et al., 2008). Attempts at DNA vaccines in humans have relied on non-specific targeting of injected DNA. Increased gene delivery through electroporation and NP delivery systems has been reported, but generally do not utilize cell-specific targeting. In one report, plasmid DNA for the nucleocapsid of severe acute respiratory syndrome coronavirus (SARS-CoV) was delivered in chitosan NPs targeted with anti-DEC-205 antibody. NP DNA delivery successfully stimulated IgG and IgA antibodies against SARS-CoV nucleocapsid, in contrast to naked DNA, which produced no detectable antibody response. In addition, DC targeting with anti-DEC-205 antibody significantly increased serum IgG against SARS-CoV nucleocapsid (Raghuwanshi et al., 2012). This approach could be translated to cancer immunotherapy as well, but consideration of particle size may be critical to induce a cytotoxic T-cell (CTL) response, given that CD8^+^ DCs are necessary to induce a CTL response and are restricted to the lymph node. Therefore, particles must drain to the lymph node, which requires a particle size of 20–200, with 40 nm being ideal (Bachmann and Jennings, 2010).

Overall, DCs are an exciting TME target for NP nucleic acid delivery; they are intimately involved in the anti-tumor response and are required for the actions of checkpoint blockade therapies. Serendipitously, their phagocytic abilities and PRRs also make them easy targets for NP delivery. These qualities have generated increased interest in NP-based vaccines, which will likely lead to several clinical trials. As multifunctional NPs are designed to deliver antigens and adjuvants to DCs, gene delivery strategies should also be considered.

### Cancer-Associated Fibroblasts

Within the tumor stroma, cancer-associated fibroblasts (CAFs) modulate tumor growth and metastasis by secreting growth factors, chemokines, and extracellular components (Kalluri and Zeisberg, 2006). In many tumors, especially desmoplastic tumors with a dense stroma, CAFs often lie between blood vessels and cancer cells. This makes CAFs an impediment to cancer-directed NP delivery (Miao et al., 2016). Cisplatin NPs with or without targeting are largely taken up by CAFs in desmoplastic pancreatic tumors (Miao et al., 2017b). Damage to fibroblasts initially reduces their supportive role and promotes tumor regression. However, chronic exposure induces expression and release of soluble factors such as Wnt16 and resistance to chemotherapy. Co-delivering Wnt16 siRNA along with cisplatin NPs can prevent resistance through this pathway (Miao et al., 2015). Since this finding, several studies have now shown that plasmid DNA can be delivered to and expressed in CAFs using lipid-based NPs. In one study, delivery of a gene that produced a soluble TNFα-related apoptosis inducing ligand (sTRAIL) to CAFs caused apoptosis in the tumor parenchyma, and ultimately tumor regression (Miao et al., 2017b). Similarly, several studies have shown that delivery of pDNA encoding “traps” can be successfully delivered to CAFs *in vivo* for cancer therapy. Traps are fusion proteins designed to be secreted and ultimately bound to soluble factors in the TME, such as chemokines and cytokines. By inhibiting these factors, metastasis and immunosuppression have been shown to be reduced, ultimately improving survival in animal models. In one report, a CXCL12 trap in combination with a PD-L1 trap promoted T-cell infiltration and reduced liver metastasis of pancreatic cancer more than either therapy alone (Miao et al., 2017a). Combination of CXCL12 and PD-L1 traps also decreased immune suppressive lymphoid structures and enhanced anti-cancer vaccine efficacy (Goodwin et al., 2017). These studies together suggest that CAFs can be used as cellular factories for production of proteins that inhibit the immunosuppressive TME. This work demonstrates the possibility that replacement or inhibition of endogenous genes in CAFs may be a feasible therapeutic strategy.

### T-cells

T-cells are important mediators of anti-tumor immunity and the targets of immune-oncology drugs such as checkpoint blockade inhibitors. The receptors PD-1 and CTLA4 expressed on T-cells promote exhaustion and thus immune evasion by cancer cells. Antibodies blocking these receptors and their ligands have proven to be effective stimulators of anti-tumor immunity and have quickly become a staple of anti-cancer therapy (Hoos, 2016). An alternative T-cell based approach to promote immune recognition of cancer cells is autologous, genetically engineered T-cells. These cells are engineered to express CARs specific to cancer epitopes using viral transduction. CAR-T cells have recently been approved by the FDA for acute lymphoblastic leukemia (ALL) and clinical trials are ongoing in many other cancer types (Landoni and Savoldo, 2017). Whether by removing checkpoint blockade or through genetic modification, T-cells have been demonstrated to be a successful target for cancer immunotherapy. New approaches to further harness the power of T-cells are being developed in many areas, including nanomedicine.

#### CAR-T-cells

Expression of CARs in primary T-cells relies on viral transduction and integration of DNA into the genome *in vitro*. Manufacturing genetically engineered cells for autologous transplantation is an intensive process with relatively low yields. T-cells are resistant to many forms of gene delivery and standard transfection protocols are not effective. Current gene delivery methods to T-cells rely on viruses or electroporation (Freeley and Long, 2013). Viral methods can be mutagenic and electroporation of cell membranes can lead to irreversible cell damage and low yields. The incorporation of efficient and transient gene expression with NP platforms to produce engineered T-cells holds promise for improved immunotherapies. Photoporation based on NPs is one such strategy. In this approach, transient permeabilization is achieved by adding gold NPs to CD8+ T-cells followed by short laser pulses, creating a photothermal effect. This strategy had lower cytotoxicity than nucleofection with comparable siRNA-mediated gene knockdown (Wayteck et al., 2017).

One potential improvement to CAR T-cell therapy is increasing the specificity of T-cells by means of removing non-cancer specific TCRs. Toward this aim, Moffett et al. report NP delivery of mRNAs to T-cells using anti-CD3 and anti-CD8 antibody targeting. Delivery of megaTAL nuclease mediated elimination of the T-cell receptor alpha constant region (TRAC), effectively removed the ability of T-cells to produce their own TCRs and resulted in the specific expression of the CAR (Moffett et al., 2017). This approach may foreseeably reduce off-target immune responses, but was not tested *in vivo*. In another approach, Moffett et al. (2017) increased the proportion of central memory T-cells, a critical cell population in establishing an effective immune response. Enrichment of the central memory T-cell phenotype was achieved by introducing Foxo1_3A_ encoding mRNA into a CD3 targeted NP platform. Treatment of T-cells with these Foxo1_3A_-encoding NPs increased the activity of CAR-modified T-cells in a mouse model of B-cell lymphoma (Moffett et al., 2017).

While these reports are intriguing, given the prevalence of viral methods in autologous T-cell therapy, it is questionable whether NP-based T-cell gene delivery will be clinically translatable. Despite greater than 200 clinical trials for CAR-T cells, none currently use NP-based methods.

#### T-cells *in Vivo*

Nanoparticle-mediated nucleic acid delivery to T-cells *in vivo* has also been demonstrated. These NP systems rely on antibodies to surface proteins expressed on T-cells. In one instance, b7 integrin targeting antibody was used to deliver lipid-based NPs containing siRNAs to leukocytes. Systemic delivery of only 2.5 mg/kg mediated gene knockdown (Peer et al., 2008). Delivery of siRNAs to CCR5, a critical receptor for HIV entry, with lymphocyte function-associated antigen 1 (LFA-1)-targeted particles decreased susceptibility of humanized mice to HIV infection (Kim et al., 2010). In these two studies, the subsets of leukocytes targeted were not described and are likely heterogeneous, considering the targeted receptors are present on many leukocytes. Alternatively, using anti-CD4 antibody decorated lipid NPs can specifically deliver siRNA to T-cells *in vivo*. Ramishetti et al. (2015) found that internalization, not endosomal escape, may be the limiting factor for T-cell gene delivery. Intriguingly, CD4 subsets with high or low CD4 expression had different rates of internalization and subsequent gene silencing (Ramishetti et al., 2015). Further research into the T-cell internalization pathways and characterization of internalization after binding to other T-cell specific receptors is warranted. Collectively, *in vivo* delivery of oligonucleotides to T-cells with NPs is achievable, but the potential therapeutic benefit for cancer is yet to be determined.

### Blood Vessels

Angiogenesis refers to the growth of new blood vessels from pre-existing vascular networks. Healthy vasculature is quiescent due to a controlled balance between pro- (e.g., VEGF and FGF) and anti- (e.g., angiostatin and thrombospondin) angiogenic factors that regulate endothelial cell proliferation and migration (Jain, 2003). As tumors outgrow their local oxygen supply, they hijack this regulation and permanently shift the balance to a pathologic, pro-angiogenic state during the “angiogenic switch” (Folkman, 1971; Hanahan and Folkman, 1996). This produces chaotic and dysfunctional vasculature. While normal blood vessels consist of a continuous monolayer of tightly adhered ECs, closely associated mural cells that promote vessel stability, and a continuous basement membrane; tumor vessels have loosely associated ECs with large gaps between them, poor mural cell recruitment, and an irregular and discontinuous basement membrane (Baluk et al., 2005). This reduced vessel wall integrity promotes leakiness and cancer cell intravasation. Thus, directly targeting tumor vessels to either inhibit their growth or promote their normalization is believed to have the potential to inhibit tumor growth and aggression, as well as metastasis (Folkman, 1971; Carmeliet and Jain, 2011). Interestingly, it is the “leaky” nature of tumor blood vessels that both makes it challenging to deliver drugs such as chemotherapy to the tumor core, but also greatly facilitates delivery of NPs to cancer cells due to the “enhanced permeability and retention effect” (Prabhakar et al., 2013).

Oligonucleotide delivery to tumor endothelium has been achieved with multiple NP platforms. Generally, successful delivery of NPs to vasculature is confirmed by visualizing co-localization of fluorescently labeled nucleic acids packaged in NPs with an endothelial stain such as the cell surface marker CD31. Chitosan NPs have been demonstrated to co-localize to both tumor and endothelial cells *in vivo* and effectively deliver siRNAs to both cell types (Lu et al., 2010). In an orthotopic model of ovarian carcinoma, treatment with chitosan NPs carrying siRNAs targeting human EZH2 (expressed in the transplanted cancer cells) or murine EZH2 (expressed in the endogenous murine vasculature) inhibited tumor growth. However, the NPs carrying murine targeting siRNA had more potent effects on inhibiting disease burden, suggesting chitosan-mediated targeting of tumor vasculature had more potent therapeutic effects than targeting cancer cells directly (Lu et al., 2010). Second-generation NPs rely on incorporation of ligands to target endothelial cell-specific surface proteins. For example, ligands to integrin αVβ3, such as the peptide RGD, can be used to facilitate NP uptake into neo-vasculature. Studies have shown that NPs containing the chemotherapeutic drug doxorubicin can be directed specifically to tumor vasculature using this ligand, causing loss of tumor blood vessels and decreased metastasis (Murphy et al., 2008). Similarly, delivery of an anti-miR to inhibit the pro-angiogenic miR-132 with these same NPs in an orthotopic xenograft mouse model of human breast cancer yielded therapeutic effects on inhibiting tumor vasculature and decreasing tumor burden (Anand et al., 2010). miRNAs have also been delivered using RGD-labeled chitosan NPs. Delivery of miR-200 family members using this approach reduced angiogenesis by direct and indirect mechanisms and resulted in reduced disease burden in ovarian cancer models (Pecot et al., 2013). RGD-chitosan mediated delivery of siRNA targeting *PLXDC1*, a growth-promoting gene, has been shown to effectively silence target gene expression in endothelial cells, with subsequent effects on promoting endothelial apoptosis and inhibiting tumor growth (Hee-Dong et al., 2010). The α_V_β_3_ integrin also facilitates uptake of viral genomic material and therefore may be an effective route for NP based gene delivery (Stewart and Nemerow, 2007). In one report, delivery of mutant *Raf-1* gene with α_V_β_3_-targeted cationic lipid NPs caused apoptosis of vessels and surrounding tumor tissues (Hood et al., 2002). Another receptor that can mediate uptake into the vascular endothelium is CD31, a classical marker of blood vessels. While αVβ3 is thought to be expressed specifically by tumor neovasculature (as well as some cancer cell types), CD31 is expressed on all endothelium (both blood, and to a lesser extent, lymphatic). Using CD31 ligands to deliver siRNAs resulted in specific decrease of target genes in vascular endothelium. By delivering siRNA to CD31 itself, tumor growth and metastasis were inhibited in a prostate cancer model (Santel et al., 2006). An alternative approach to ligand-based targeting is chemically modified dendrimers that can specifically target the endothelium (Khan et al., 2015). 7C1 NPs are another type of NP that have been reported to localize faithfully and specifically to the endothelium in multiple models of aberrant vascular function, including tumor angiogenesis. These NPs are able to elicit at least 50% knockdown of target endothelial gene expression, and simultaneously deliver siRNAs targeting multiple genes in the endothelium (Dahlman et al., 2014).

## Summary

In an era where clinical trials in nucleic acid delivery have become a reality, we can expand our scope to consider new and exciting gene and cell targets for cancer therapy. NP uptake by cells within the TME has traditionally been considered a delivery obstacle for NP-based systems, however, turning TME cells into targets could lead to new therapeutic strategies. Biology has taught us that non-transformed cells can act as accessories to cancer growth and spread, but that strategies to reprogram cells in the TME could result in revolutionary therapies. The studies highlighted in this review demonstrate NP-based nucleic acid delivery strategies for reprograming the TME. In effect, turning the TME from a permissive space for cancer growth to a hostile one. This strategy is synergistic with current immunotherapy and anti-angiogenic approaches and could feasibly extend the efficacy of these paradigm-shifting treatments.

## Author Contributions

All authors listed have made a substantial, direct and intellectual contribution to the work, and approved it for publication.

## Conflict of Interest Statement

The authors declare that the research was conducted in the absence of any commercial or financial relationships that could be construed as a potential conflict of interest.
